# Research Progress on Rashba Effect in Two-Dimensional Organic–Inorganic Hybrid Lead Halide Perovskites

**DOI:** 10.3390/nano14080683

**Published:** 2024-04-16

**Authors:** Junhong Guo, Jinlei Zhang, Yunsong Di, Zhixing Gan

**Affiliations:** 1 College of Electronic and Optical Engineering & College of Flexible Electronics (Future Technology), Nanjing University of Posts and Telecommunications, Wenyuan Road 9, Nanjing 210023, China; jhguo@njupt.edu.cn; 2 School of Physical Science and Technology, Suzhou University of Science and Technology, Suzhou 215009, China; zhangjinlei@usts.edu.cn; 3 Center for Future Optoelectronic Functional Materials, School of Computer and Electronic Information, Nanjing Normal University, Nanjing 210023, China; 4 College of Materials Science and Engineering, Qingdao University of Science and Technology, Qingdao 266042, China

**Keywords:** Rashba effect, photoluminescence, 2D perovskites, optoelectronics and spintronics

## Abstract

The Rashba effect appears in the semiconductors with an inversion–asymmetric structure and strong spin-orbit coupling, which splits the spin-degenerated band into two sub-bands with opposite spin states. The Rashba effect can not only be used to regulate carrier relaxations, thereby improving the performance of photoelectric devices, but also used to expand the applications of semiconductors in spintronics. In this mini-review, recent research progress on the Rashba effect of two-dimensional (2D) organic–inorganic hybrid perovskites is summarized. The origin and magnitude of Rashba spin splitting, layer-dependent Rashba band splitting of 2D perovskites, the Rashba effect in 2D perovskite quantum dots, a 2D/3D perovskite composite, and 2D-perovskites-based van der Waals heterostructures are discussed. Moreover, applications of the 2D Rashba effect in circularly polarized light detection are reviewed. Finally, future research to modulate the Rashba strength in 2D perovskites is prospected, which is conceived to promote the optoelectronic and spintronic applications of 2D perovskites.

## 1. Introduction

Organic–inorganic hybrid lead halide perovskites (OILHPs) have attracted significant interest in the past years due to their outstanding performance as solar absorbers in photovoltaics [1,2,3,4,5]. The long carrier lifetime of photogenerated carriers is a crucial factor for excellent optoelectronic performance [6]. An extraordinarily long carrier lifetime (*τ* ≥ 1 μs) and a substantial carrier diffusion length (*L_D_* ≥ 5 μm) have been measured in polycrystalline perovskite thin films with moderate mobility (*μ* ≈ 1–100 cm^2^ V^−1^ s^−1^), which is drastically lower than that of other conventional semiconductors, such as GaAs (*μ* ≈ 500 cm^2^ V^−1^ s^−1^). However, the physical mechanism behind the long carrier lifetime is still elusive [7,8,9,10,11]. The mainstream investigation attributes it to the low trap density [12,13], which may lead to a significant suppression in nonradiative recombination, thus greatly prolonging the carrier’s lifetime. However, further research has found that, in perovskites with relatively high defect density, the carrier lifetime does not significantly decrease [14]. Therefore, the correlation between carrier lifetime and defect density in perovskite is not definite. Currently, other models, such as high defect tolerance [15,16,17], photon recycling [18,19], weak electron–phonon coupling [20,21,22,23], the presence of ferroelectric domains [24,25], the formation of polarons, and the screening of band-edge charges [26], have been proposed to rationalize the long carrier lifetime of perovskites. However, after years of laborious exploration, there are still some inherent limitations and inconsistencies in the above-mentioned models.

**Figure 1:**
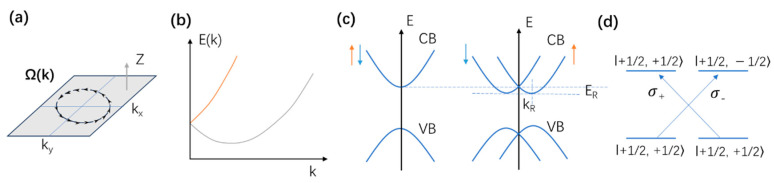
(**a**) Effective magnetic field Ω(**k**) induced by the Rashba effect showing the variation of the direction at a fixed value of |**k**|. (**b**) Energies of the spin eigenstates as a function of the in-plane wave vector. (**c**) The electron dispersion relation shows a doubly degenerate parabolic band at k = 0 subject to Rashba spin splitting, fostering two parabolic bands with opposite spins (arrows). (**d**) Optical selection rules for interband transitions at the band gap.

Among them, the Rashba effect is also considered to be one of the most essential reasons for the long carrier lifetime [27,28,29]. The Rashba effect was proposed in the 1950s, which reveals spin splitting in noncentrosymmetric semiconductors [30,31]. For ordinary semiconductors, the dispersion of the conduction band minimum (CBM) electrons and valence band maximum (CBM) holes can be described as a spin-degenerate parabolic energy band,

(1) $$E\left(k\right)=\hbar^{2}k^{2}/2m^{*}$$

where ***k*** is the electron wavevector, ℏ is the reduced Planck constant, and m* is the effective mass of electrons (or holes). However, if the semiconductor lacks inversion symmetry, and meanwhile there is strong spin-orbit coupling, an effective magnetic field ***Ω****(****k****)* appears (Figure 1a), which lifts the degeneracy of the carrier spin states within each band [32]. Thus, when the Rashba effect occurs, the spin-degenerate band splits into two spin-polarized sub-bands deviating from the symmetric center of the Brillouin zone (Figure 1b,c).

(2) $$E_{\pm }\left(k\right)=\hbar^{2}k^{2}/2m^{*}\pm \alpha_{R}\left|k\right|$$

α_R_ is the Rashba splitting constant.

(3) $$\alpha_{R}=\frac{2E_{R}}{k_{R}}$$

Figure 1c shows that the Rashba effect has two important characteristics, namely, energy band splitting and in-plane spin splitting. *E_R_* and *k_R_* are the energy difference and momentum offset between the vertex of the energy curve and the k origin at the high-symmetry point, respectively. The strength of the Rashba effect is usually characterized by the Rashba constant *α_R_*.

Due to the different orbital compositions of the conduction band and valence band, the splitting degrees of the CBM and VBM are not equal. Therefore, the splitting will make the carrier recombination in perovskite exhibit features similar to indirect bandgap, thereby reducing the carrier recombination rate. In addition, because the conduction band and valence band have opposite spin helicity, carrier recombination is spin forbidden, which further reduces the electron-hole recombination rate. Optical selection rules for interband transitions at the band gap are plotted in Figure 1d. The Rashba effect not only provides a possible explanation for the long carrier lifetime in perovskites but also enables effective control and manipulation of the polarized spins in spintronic devices. Apart from the research on conventional optoelectronics areas, such as solar cells, LEDs, and photodetectors [33,34,35,36], one of the exciting research directions on lead halide perovskites would be spintronics-related technology.

The Rashba effect is considered one of the most essential reasons for the long carrier lifetime of OILHPs. Moreover, the photoelectronic properties of OILHPs that can be regulated by the magnitude of the Rashba effect. Thus, the Rashba effect in 2D OILHPs has attracted increasing research interest. In this mini-review, the recent research progress on the Rashba effect in 2D perovskites is summarized. Several important aspects of the Rashba effect in 2D perovskites, including the origin and magnitude, layer dependence in 2D perovskite, the Rashba effect in 2D perovskite quantum dots, 2D/3D composite, and van der Waals heterostructures, are included. In addition, circularly polarized light-detection applications based on the Rashba effect are discussed. Due to the limitation of scope, this review does not include all achievements related to this topic, and only a selection of representative examples is discussed. We hope this mini-review can further stimulate research enthusiasm on this important topic so that more insights into the fundamental understanding can be gained and more optoelectronic and spintronic applications can be developed.

## 2. Rashba Effect in Three-Dimensional (3D) Perovskites

It is generally believed that the crystal structure of three-dimensional OILHP (such as MAPbI_3_, MA = methylamine) at room temperature is a tetragonal system (or cubic system or orthogonal system, depending on the material composition) with centrosymmetry. However, it has been found that the perovskite lattice does not have strict centrosymmetry [29]. The lead halide octahedron [MX_6_]^4−^ in the perovskite lattice is slightly distorted [37], and the organic cation A^+^ also has a certain orientation in a rapidly rotating state [38,39]. These properties may disrupt the centrosymmetry of the perovskite lattice. In addition, there is strong spin-orbit coupling due to the presence of heavy elements, such as lead, tin, and iodine. The Rashba effect in perovskites is expected to be strong. Based on the above reasons, many theoretical studies predict a strong Rashba effect in perovskites [37,40]. For example, the spin-orbit coupling in MAPbI_3_ causes a displacement of the conduction band energy level of more than 1 eV [41]. In addition, a few experimental studies also strongly support the occurrence of the Rashba effect in the compound [42,43]. A significant effort regarding the experimental observation of Rashba spin-splitting has been demonstrated by Giovanni and co-workers through spin-dependent circularly polarized pump-probe experiments [42]. Neisner et al. directly observed the split in the valence band by angle-resolved photoemission spectroscopy measurements [43].

## 3. Rashba Effect in Two-Dimensional Perovskites

Two-dimensional OILHPs are commonly known as the Ruddlesden–Popper (RP) phase [44,45,46,47,48,49,50,51] and the Dion–Jacobson (DJ) phase [52]. Taking RP-phase 2D perovskite as an example, its general chemical structure is (RNH_3_)_2_A_n−1_M_n_X_3n−1_ (*n* = 1, 2, 3, 4…), where RNH_3_ is usually an organic group of aliphatic or aromatic alkylammonium, such as 2-phenylethylammonium (PEA) and n-butylammonium (n-BA), A is a monovalent organic cation, such as CH_3_NH_3_^+^ (abbreviated as MA^+^) and HC(NH_2_)_2_^+^ (abbreviated as FA^+^), and M is a divalent metal cation, mainly referring to lead Pb, X is a halide anion. The large organic cations (RNH_3_^+^) separate the layers of the inorganic Pb-I network. And *n* represents the number of inorganic [MX_6_]^4−^ octahedral structures in each period. The 2D OILHPs have attracted increasing research interest due to their special multi-quantum-well structures and excellent structural stability under ambient conditions [53,54].

**Figure 2:**
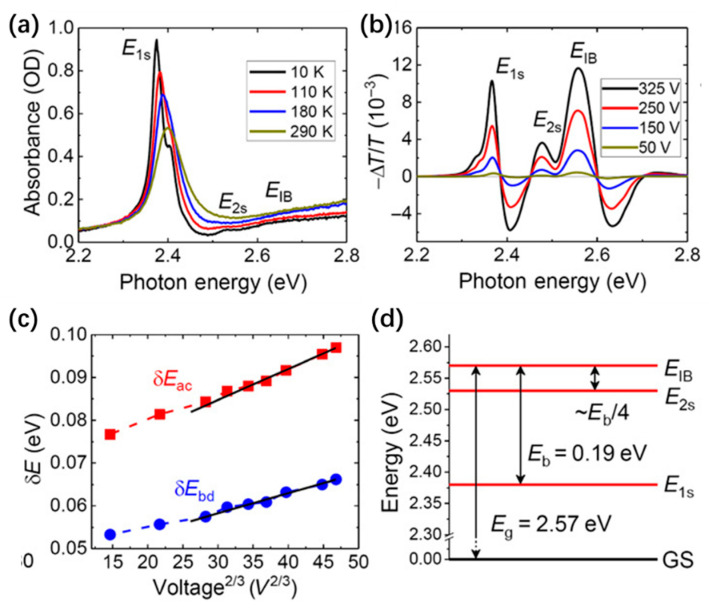
(**a**) Absorption spectra of (C_6_H_5_C_2_H_4_NH_3_)_2_PbI_4_ film at various temperatures, which contains 1s and 2s exciton (labeled as E_1s_ and E_2s_, respectively) and an interband (IB) transition. (**b**) Electroabsorption spectra of (C_6_H_5_C_2_H_4_NH_3_)_2_PbI_4_ thin film measured at 45 K at various applied to electric fields. (**c**) Energy differences δE_ac_ and δE_bc_ plotted versus V^2/3^. (**d**) Energy levels of the excitons and interband transition (IB) with respect to the ground state (GS) [59]. Reproduced with permission under Creative Common CC-BY 4.0 license.

Bychkov and Rashba proposed that the Rashba effect also appears in two-dimensional (2D) electron gas systems [55]. Thus, the Rashba effect has been extensively investigated in various 2D material systems, including III−V semiconductor heterostructures and topological insulators Bi_2_Se_3_ over the past few decades [56,57,58]. Nevertheless, Rashba splitting energy in these 2D structures is typically smaller than 10 meV, limiting the performance of spintronic devices based on these 2D materials [56,57,58]. The Rashba effect in 2D perovskites has also attracted extensive research attention. Density functional theory (DFT) calculation is an important tool for defining and demonstrating the existence of Rashba splitting, as well as quantifying the structural symmetry, rotation, and distortion. For example, Zhai et al. showed the existence of Rashba splitting in the plane perpendicular to the 2D layer of (C_6_H_5_C_2_H_4_NH_3_)_2_PbI_4_ based on the DFT calculations using local density approximation (LDA) in the form of ultrasoft pseudopotentials [59]. In more detail, the first-principles DFT calculations show that the breaking of inversion symmetry is caused by the displacement of the Pb atom from the octahedra center, which leads to the Rashba splitting. At temperatures below 110 K, the absorption spectrum in the photon energy range of 2.45 to 2.65 eV shows two step-like absorption edges, which are assigned as the 1s and 2s exciton energy at 2.38 and 2.53 eV, respectively (Figure 2a). Considering the band edge of (C_6_H_5_C_2_H_4_NH_3_)_2_PbI_4_ at 2.57 eV (Figure 2b), 1s and 2s exciton binding energies are about 190 ± 4 meV and 45 ± 8 meV, respectively. The energy differences between *δE_ac_* and *δE_bc_* scales with *V*^2/3^ indicate a Frank–Keldysh-type oscillatory feature at the continuum band edge (Figure 2c). From the electroabsorption spectrum and photoinduced absorption spectra of excitons and free carriers, they obtained a giant Rashba splitting in 2D (C_6_H_5_C_2_H_4_NH_3_)_2_PbI_4_ thin film, with energy splitting of (40 ± 5) meV and a Rashba constant of (1.6 ± 0.1) eV·Å (Figure 2d) [59].

In addition, Todd et al. investigated carrier dynamics in 2D (BA)_2_MAPb_2_I_7_ thin film by time-resolved circular dichroism techniques [60]. They revealed the presence of a Rashba spin splitting via the dominance of processional spin relaxation induced by the Rashba effective magnetic field. The Rashba spin-splitting magnitude was extracted from simulations of the measured spin dynamics incorporating longitudinal optical-phonon and electron–electron scattering, yielding a value of 10 meV at an electron energy of 50 meV above the band gap, which is twenty times larger than that in GaAs quantum wells. Moreover, a Rashba splitting of 85 meV with a Rashba coefficient α_R_ of 2.6 eV Å was observed in an emergent 2D DJ phase (AMP)PbI_4_ (AMP = 4-(aminomethyl)piperidinium) [61]. Jana et al. introduced a structural chirality transfer across the organic–inorganic interface in 2D perovskites using appropriate chiral organic cations [62]. The chiral spacer cations and their asymmetric hydrogen-bonding interactions with lead bromide-based layers cause symmetry-breaking helical distortions in the inorganic layers. The first-principles calculation predicts a substantial bulk of the Rashba–Dresselhaus spin-splitting in the inorganic-derived conduction band with opposite spin textures between R- and S-hybrids due to the broken inversion symmetry and strong spin-orbit coupling. The chirality transfer from one structural unit to another represents a promising approach to breaking symmetry that modulates the Rashba effect for spintronics and related applications. These findings indicated that 2D hybrid perovskites have great potential for applications in spintronics.

### 3.1. Origin and Magnitude of Rashba Spin Splitting in 2D RP Perovskites

Rashba spin splitting has been observed in multiple 2D OILHPs, yet with a significant variance in the magnitude of spin splitting [58,59,60,61]. However, the origin of the giant Rashba splitting remains elusive. The crucial role of the orientation of the organic cation in the 2D RP perovskite was explored by Kagdada et al. Their DFT calculation results revealed that the MA cation rotation imposes structural distortion in the inorganic PbI_6_ layer, which then varies the structure and value of the electronic bandgap, charge density, and optical absorption. The strong spin–orbit coupling leads to a wide range of Rashba splitting parameters from 0.04 to 0.278 eV Å. The simulated optical absorption spectra showed that absorption edges for the different orientations of the MA molecule are not the same [63].

**Figure 3:**
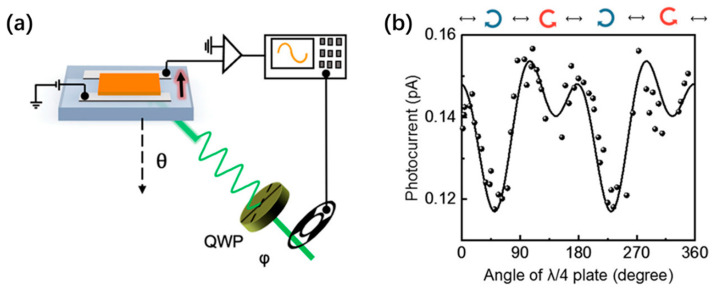
(**a**) Schematic illustration of the experimental setup for measurement of photogalvanic current. The φ indicates the angle between the fast axis of the quarter-wave plate (QWP) and the incident light polarization. The θ indicates the incident angle of excitation light. (**b**) Room temperature photogalvanic current of (AMP)PbI_4_ versus QWP rotation angle φ, measured at θ = 60° and excited via a 556 nm continuous laser [64]. Reproduced with permission. Copyright 2021, American Chemical Society.

In addition, Zhou et al. obtained (AMP)PbI_4_ DJ phase crystals by an economical aqueous method. They clarified the origin of the giant Rashba effect by temperature- and polarization-dependent photoluminescence (PL) results [64]. The strong temperature-dependent PL helicity indicates the thermally assisted structural distortion as the main origin of the Rashba effect, suggesting that valley polarization still preserves at high temperatures. The Rashba effect was further confirmed by the circular photogalvanic effect near the indirect bandgap (Figure 3).

In addition, organic–inorganic hybrid halide perovskites are susceptible to dynamic instabilities known as octahedral tilt, which involves a rigid rotation of the inorganic octahedral cages and can occur along any of the three Cartesian directions in the crystal with either in-phase or out-of-phase ordering [65]. While the phase transitions related to octahedral tilt have been thoroughly examined in 3D hybrid halide perovskites, their influence on hybrid 2D perovskites remains not fully comprehended. To gain insight into this puzzle, Shao et al. utilized scanning tunneling microscopy to directly visualize the surface octahedral tilt in freshly exfoliated 2D RP perovskites across the homologous series [66]. The steric hindrance imposed by long organic cations is unlocked by exfoliation. The experimentally determined octahedral tilts from 2D RP-phase perovskites of *n* = 1 to *n* = 4 align closely with the out-of-plane surface octahedral tilts predicted by DFT calculations. The out-of-plane octahedral tilt of the exfoliated surface is correlated to the redshifted emission peak alongside the primary exciton in the PL spectra. Therefore, the Rashba spin splitting is attributed to the octahedral tilt [66].

### 3.2. Layer-Dependent Rashba Band Splitting in 2D Perovskites

It is very significant to reveal the impacts of surface termination and the number of inorganic layers on the amplitude of Rashba band splitting so as to enhance the understanding of the origin and extent of Rashba spin splitting in 2D RP-phase perovskites. Thus, research efforts were devoted to the layer-dependent Rashba band splitting in 2D perovskites. Singh et al. investigated Rashba spin splitting in 2D RP (BA)_2_(MA)*_n_*_−1_Pb*_n_*I_3_*_n_*_+1_ with both centrosymmetric (*n* = 1) and noncentrosymmetric (*n* = 2 and 3) structures, using first-principle calculations, polarization, and temperature-dependent PL spectroscopy [67]. They revealed the *n*-dependent Rashba spin splitting in 2D RP perovskites. When *n* = 1, a single metal halide octahedral layer is sandwiched between long BA^+^ organic cations, Rashba spin splitting is the largest. As *n* increases, the Rashba spin splitting decreases. The large Rashba effect observed in the 2D RP perovskite of an *n* = 1 structure is attributed to the local distortion of the PbI_6_ octahedron at the surface [67].

**Figure 4:**
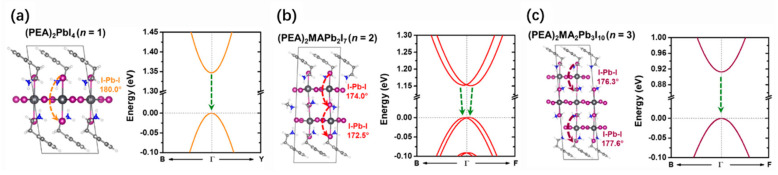
(**a**) Optimized crystal structures and electronic band structures of (**a**) (PEA)_2_PbI_4_ (*n* = 1), (**b**) (PEA)_2_MAPb_2_I_7_ (*n* = 2), and (**c**) (PEA)_2_MA_2_Pb_3_I_10_ (*n* = 3) [68]. Reproduced with permission. Copyright 2018, American Chemical Society.

By using a combination of DFT calculations and time-resolved PL spectroscopy, Yin et al. compared the Rashba band splitting of the prototype 3D MAPbI_3_ and the 2D RP perovskites [68]. They demonstrated that significant structural distortions associated with different surface terminations are responsible for the observed Rashba effect in 2D OILHPs. Interestingly, their calculation results indicated that the intrinsic Rashba splitting occurs in the perovskite crystals with an even number of inorganic layers (*n* = 2), in consistency with their longer PL lifetimes and ground-state bleaching recovery lifetimes. Whereas, when the number of inorganic layers is odd (*n* = 1 and *n* = 3), the Rashba effect of 2D RP perovskites absences (Figure 4). These findings elucidate the significant impact of the number of inorganic layers on the electronic properties of 2D perovskites, suggesting the controlling of the *n* value in 2D RP perovskites to design Rahsba effects for spintronic applications.

In addition, Liu investigated the thickness-dependent structural distortion along with the Rashba splitting energy by using the DFT calculation [69]. Three types of OILHPs were compared to explore the effect of halogens and organic ligands. As the thickness increases, the structural distortion degree decreases. The Rashba splitting magnitude follows the same tendency. The 2D MAPbI_3_ is less sensitive to thickness change compared to the 2D MAPbBr_3_ or the 2D MAPbCl_3_. Furthermore, ligands and their orientations have dramatically different impacts on the Rashba splitting. The PEA ligands enhance the Rashba splitting magnitude, while the BA ligands have the converse effect. The partial charge-density analysis shows that the band edges are contributed to by a charge density at a specific layer in the structure. Thus, they concluded that the Rashba effect is layer dependent in 2D HOIPs [69].

### 3.3. Rashba Effect in 2D Perovskite Quantum Dots

Because of the quantum confinement effect, the quantum dot usually shows fast radiative recombination, large exciton binding energies [70], and giant oscillator transition strengths [71]. Most theoretical descriptions of the Rashba effect on exciton fine structures were conducted in the weak-confinement regime, in which the exciton Bohr radius, $r_{B}$, is much smaller than the typical size of the nanocrystals. The Rashba effect was treated perturbatively, which is a valid approach, assuming $\alpha k\ll \frac{\hbar^{2}k^{2}}{2m^{*}}$, where α_e_ and α_h_ are the Rashba coefficients in the conduction and valence bands, respectively, ***k*** is the typical quasi-momentum of exciton center-of-mass (COM) motion, and $m^{*}$ is the effective mass of the COM motion. The momentum is ***k*** ∼ 1/R for an exciton confined in an NC with size R, so the perturbative approach is valid when $\alpha \ll \hbar^{2}/2m^{*}R$. This condition is clearly not satisfied in a large NC ($R\gg \hbar^{2}/2m^{*}\alpha$) or in NCs with enormously large Rashba coefficients. Thus, the Rashba effect in 2D perovskite quantum dots is elusive. To explore this question, Swift et al. constructed an effective mass model of excitons in 2D perovskite quantum dots, which covers the full range of NC sizes and Rashba strengths [72]. The fine structure and oscillator transition strengths of Rashba excitons confined in a 2D cylindrical quantum dot are quite unusual. One notable aspect of the energy-level structure is the proliferation of dark exciton states. These dark states in large quantum dots are also likely to be thermally populated even at quite low temperatures, reducing the radiative decay rate and, consequently, the PL quantum yield of these structures.

### 3.4. Rashba Effect in 2D/3D Composite Perovskite Films

Compared with common 2D perovskite, the 2D/3D composite perovskite may have a variety of gains, such as significant interface asymmetry and an effective energy-transfer process. On the one hand, the interface asymmetry can enhance the band splitting. On the other hand, energy transfer can be used to improve the photoresponse. These two effects make 2D/3D composite perovskite promising for opto-spintronic applications. The recent development of chiral 2D/three-dimensional (3D) composite perovskites offers a new opportunity to engineer the Rashba effect. Li et al. synthesized one pair of chiral 2D/3D composite perovskite [73]. The optical properties were studied by polarization-dependent femtosecond transient absorption (fs-TA) spectroscopy, which revealed that the chiral properties of organic cations were successfully transferred to the achiral part. The Rashba effect is significantly enhanced in the 2D/3D composite structures. The spintronic relaxation along with the Rashba effect in the 2D/3D composite structures will inspire the further development of the next generation of opto-spintronic devices.

### 3.5. Rashba Effect of Van Der Waals Heterostructures Based on 2D Perovskites

**Figure 5:**
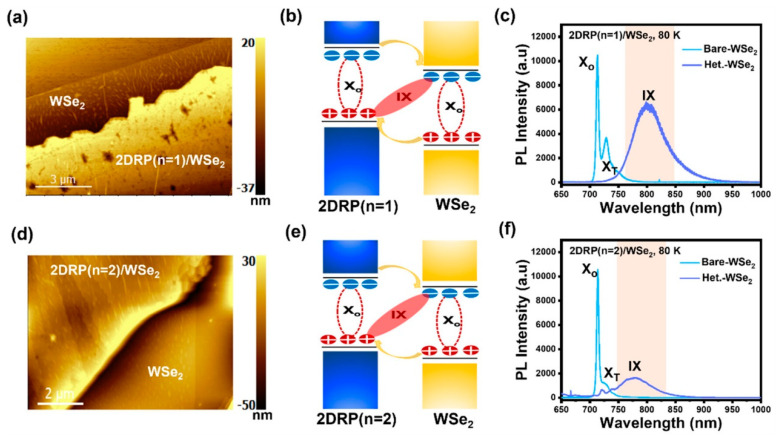
Topography (**a**,**d**), band structures (**b**,**e**), and PL spectra (**c**,**f**) of 2D RP (*n* = 1)/WSe_2_ (**a**–**c**) and 2D RP (*n* = 2)/WSe_2_ heterostructures (**d**–**f**) under 633 nm excitation. The type-II band alignment between monolayer WSe_2_ and 2D RP perovskites leads to the formation of interlayer excitons [67]. Reproduced with permission. Copyright 2023, American Chemical Society.

The van der Waals heterostructures based on different 2D materials enable innovative device engineering. A variety of van der Waals heterostructures have been developed based on 2D perovskites for optoelectronic applications. Thus, it is very significant to investigate the Rashba effect in van der Waals heterostructures. Singh et al. integrate an RP-phase 2D perovskites monolayer with another important family of 2D excitonic semiconductors, i.e., transition-metal dichalcogenides (TMDs) [67]. A combined effect of Rashba spin splitting in 2D RP perovskites and the strong spin–valley physics of monolayer TMDs can give rise to effective spin–valley polarization in the heterostructures using circularly polarized light (CPL) excitation. Thus, the 2D RP perovskite/TMD heterostructure provides an attractive material combination for investigating valleytronic phenomena, as it reduces fabrication complexity and sample-to-sample variance. Different 2D RP perovskites (*n* = 1 and 2) and monolayer WSe_2_s were coupled to form 2D vdW heterostructures. Robust interlayer excitons (IXs) in staggered type-II band-aligned heterostructures were observed (Figure 5). These IXs are strongly valley-polarized with exciton lifetimes longer than the intralayer excitons in the constituent monolayer TMDs, suggesting the spin–valley-dependent optical selection rules to the IXs. This research broadens the scope for exploring spin–valley physics in heterogeneous stacks of 2D semiconductors. They also investigated a 2DRP-(*n* = 1)/MoS_2_ heterostructure with a broken type-III band alignment. In contrast, there is no interlayer charge transfer, thus the 2DRP/MoS_2_ heterostructure does not show any IX emission.

### 3.6. Applications of 2D Rashba Effect in Circularly Polarized Light Detection

CPL is a special light beam, which consists of two spiral modes called chirality or handedness. Based on the rotation of the field vector, the CPL can either rotate counterclockwise (left handed, *σ*^+^) or clockwise (right handed, *σ*^−^) when observed from the direction opposite to the wave’s propagation. Direct detection of CPL is a challenging task due to limited materials and ambiguous structure–property relationships that lead to low distinguishability of the light helicities. On the one hand, the circular photogalvanic effect is considered the most important experiment that confirms the presence of the Rashba effect in semiconductors. The circular photogalvanic effect has been demonstrated in a variety of materials with the Rashba effect, such as GaAs/AlGaAs multi-quantum wells, the polar semiconductor BiTeI, 2D transition-metal dichalcogenides, and topological insulators [74,75,76,77]. On the other hand, the Rashba effect in 2D perovskites provides new opportunities for dealing with the challenge of CPL detection.

**Figure 6:**
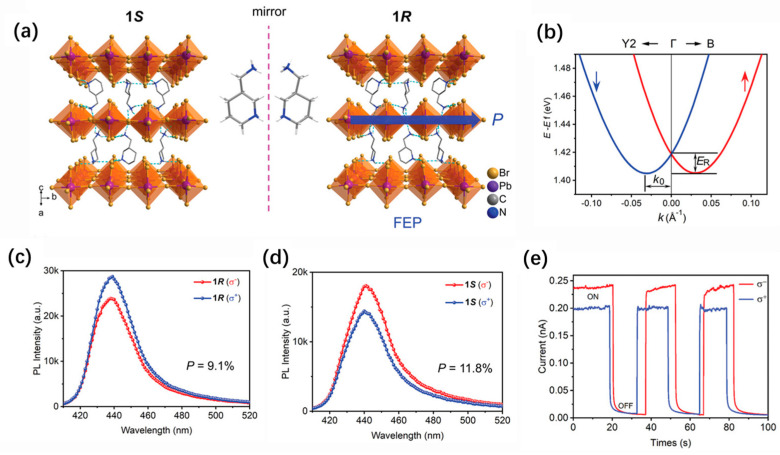
(**a**) Crystal structures of (S-3AMP)PbBr_4_ (1S) and (R-3AMP)PbBr_4_ (1R) in the ordered ferroelectric phase (FEP). (**b**) Rashba splitting band structure of 1R. (**c**,**d**) CPLEPL spectra of 1R (**c**) and 1S (**d**) upon L-CPL (σ^+^) and R-CPL (σ^−^) excitation at 395 nm. (**e**) Photocurrent differences upon L- and R-CPL irradiation at 430 nm [86]. Reproduced with permission. Copyright 2022, Wiley-VCH.

Chiral 2D perovskites have been recently explored as the responsive component for the direct detection of CPL [78,79,80,81,82,83,84]. For example, Wang et al. inserted chiral organic ligands into the organic layers of 2D perovskites to obtain chiral (R-MBA)_2_PbI_4_ and (S-MBA)_2_PbI_4_. The in-plane photocurrent response generated by the CPL excitation of planar photoconductive devices shows a typical response of the chirality-induced circular photogalvanic effect that originates from the Rashba splitting in the electronic bands of these compounds, demonstrating the potential applications of chiral 2D perovskites in optoelectronic devices that are sensitive to the light helicity [85]. Similarly, Fan et al. report direct CPL detection by using a pair of 2D chiral perovskite ferroelectrics, (*R*/*S*-3AMP)PbBr_4_ (3AMP = 3-(aminomethyl)-piperidine divalent cation) [86]. These 2D perovskites undergo a phase transition at 420 K that is a combination of order–disorder and displacive ferroelectric transition. DFT calculations and circularly polarized light-excited PL measurements have confirmed the presence of the Rashba effect in these 2D chiral perovskites (Figure 6a–d). This effect results in spin selectivity, which can modulate the behavior of photogenerated charge carriers during transitions, recombination, and transfers. Single-crystal-based devices have been shown to directly detect CPL at 430 nm, with an on–off ratio of current higher than 1.7 × 10^3^ and anisotropy factors of responsivity larger than 0.20 (Figure 6e). The enhanced CPL detection is attributed to the Rashba effect, which has a large Rashba coefficient of 0.93 eV·Å.

## 4. Conclusions and Outlook

In summary, this mini-review focuses on the Rashba effect in 2D perovskites. Recent research progress on the origin and extent of Rashba spin splitting, layer-dependent Rashba band splitting of 2D perovskites, the Rashba effect on 2D perovskite quantum dots, the Rashba effect in 2D/3D composite perovskite, and the Rashba effect in van der Waals heterostructures based on 2D perovskites are reviewed. In addition, applications of the 2D Rashba effect in circularly polarized light detection are included in this review.

Despite considerable reports on Rashba effects in 2D perovskites, the origin of Rashba spin splitting in 2D perovskites is still under debate. Future research efforts to investigate the impacts of the surface termination, the number of inorganic layers, the structure of organic spacers, the planar sizes, and the distortion of inorganic octahedrons on the magnitude of Rashba band splitting will not only gain more insight into the origin of Rashba effect in 2D perovskites but also inspire approaches to modulate the Rashba spin splitting. In addition, the relationship between charge-carrier dynamics and the Rashba effect in 2D perovskites is still to be established, so that the photoelectronic properties and photophysics of 2D perovskites can be effectively controlled by modulating the Rashba magnitude. Apart from the research on conventional optoelectronics areas, such as solar cells, LEDs, and photodetectors, one of the exciting research interests on 2D perovskites will be focused on spintronics-related technology. However, the current related research is still insufficient. In other words, there is plenty of room to design new spintronic devices based on 2D perovskites.
